# Elevated diurnal CD36 expression disrupts the bile acid synthesis rhythm leading to cholestatic liver injury and inflammation via the HMGCR/CYP7A1 axis

**DOI:** 10.1016/j.gendis.2025.101776

**Published:** 2025-07-23

**Authors:** Yang Zhang, Mingyang Zhang, Shuning Fu, Zhenyu Wang, Yunfei Zhao, Junhua Gong, Miao Chen, Nuo Zhang, Mengyue Chen, Xiong Z. Ruan, Yaxi Chen

**Affiliations:** a Centre for Lipid Research & Chongqing Key Laboratory of Metabolism on Lipid and Glucose, Key Laboratory of Molecular Biology for Infectious Diseases (Ministry of Education), Institute for Viral Hepatitis, Department of Infectious Diseases, The Second Affiliated Hospital, Chongqing Medical University, Chongqing 400016, China; b John Moorhead Research Laboratory, Centre for Nephrology, University College London Medical School, Royal Free Campus, University College London, London NW3 2PF, United Kingdom; c Chongqing Key Laboratory for Pharmaceutical Metabolism Research, Chongqing Pharmacodynamic Evaluation Engineering Technology Research Center, College of Pharmacy, Chongqing Medical University, Chongqing 400016 China; d Department of Hepatobiliary Surgery, The Second Affiliated Hospital of Chongqing Medical University, 400016 Chongqing, China

**Keywords:** Bile acid metabolism, CD36, Cholestatic liver injury, Circadian rhythm, Liposomal delivery

## Abstract

Cholestatic liver diseases, including primary biliary cholangitis (PBC) and primary sclerosing cholangitis (PSC), are characterized by disrupted bile acid (BA) homeostasis and subsequent liver injury. Emerging evidence indicates that circadian rhythms significantly influence liver metabolism and the pathogenesis of liver diseases. CD36 has been identified as a regulator of the hepatic circadian clock and metabolic processes; however, the specific mechanisms by which CD36 links circadian rhythms to cholestatic liver disease remain unclear. In this study, we employed bile duct ligation (BDL) mice and liver-specific CD36 knockout (CD36 LKO) mice to examine the role of CD36 in BA metabolism and circadian gene expression. BDL mice presented disrupted rhythms in both liver clock and BA metabolism, accompanied by increased diurnal expression of CD36. Conversely, in the context of BDL, CD36 LKO reduced cholestatic liver injury, improved BA metabolism, and restored diurnal variation of BA levels. Transcriptomic analysis revealed that BA metabolism genes were regulated by CD36, particularly those involved in synthesis, which displayed diurnal variation. Targeted inhibition of CD36 expression effectively mitigated liver injury and inflammation in BDL mice by restoring the rhythmicity of HMGCR/CYP7A1 and normalizing the BA pool size. These findings suggest that CD36 plays a pro-cholestatic role through its regulation of rhythmic BA synthesis and that its inhibition may represent a promising therapeutic strategy for cholestatic liver diseases.

## Introduction

Cholestatic liver disease is characterized by toxic bile acid (BA) accumulation, which triggers a cascade of inflammation and metabolic remodeling, affects liver function, and often progresses to fibrosis and ultimately liver failure.^1^ Primary biliary cholangitis (PBC) and primary sclerosing cholangitis (PSC) are the predominant chronic cholestatic liver diseases, and their prevalence and incidence have increased in recent years.^2^ The pathogenesis of PSC and PBC is intricate, and the present therapeutic approaches primarily aim to modify the BA pool.^3^ However, two such medications, ursodeoxycholic acid, and obeticholic acid, have shown limited efficacy in many patients and are associated with adverse outcomes.^4^^,^^5^ Therefore, there is a substantial need to explore the potential mechanisms and innovative therapies to improve the prognosis of PBC and PSC patients.

In mammals, the circadian clock is an internal biological system consisting of transcriptional and translational loops of circadian clock genes that are ubiquitously expressed and interconnected within the cell, which aligns physiological processes and behaviors with day/night cycles.^6^ Circadian rhythms regulate diverse physiological events, including metabolism in all organisms. As the largest metabolic organ, the liver orchestrates multiple metabolic pathways and is tightly controlled by the biological clock.^6^ Research indicates a dynamic interplay between BAs and circadian rhythms, which collaboratively influence energy homeostasis and metabolic regulation.^7^, ^8^, ^9^ Disruption of circadian rhythms has been shown to interfere with BA metabolism, thereby increasing the risk of metabolic disorders. Conversely, abnormalities in BA signaling can disturb circadian rhythms, contributing to the development of diverse pathological conditions.^10^ Despite extensive studies supporting the connection between BAs and the circadian rhythm, the changes in and role of the circadian rhythm in cholestatic liver disease have not been thoroughly elucidated to date, which could be a breakthrough.

The metabolic homeostasis of BAs relies on their synthesis, transport, decomposition, and other pathways, as well as the coordinated function of various organs. Research indicates that mutations or inhibition of key genes involved in BA metabolism can promote the onset and progression of PBC and PSC.^11^, ^12^, ^13^ Notably, abnormal BA synthesis is a significant pathogenic factor in cholestatic liver disease. The synthesis of BAs, initiated by the rate-limiting enzyme cholesterol 7α-hydroxylase (CYP7A1) and catalyzed by a series of enzymatic reactions, is regulated by the farnesoid X receptor (FXR) and small heterodimer partner (SHP) through a feedback mechanism that inhibits BA biosynthesis.^14^ Interestingly, BA synthesis exhibits distinct diurnal rhythms, with key enzymes such as CYP7A1 and CYP8B1 displaying robust diurnal patterns that synchronize with feeding cycles and metabolic demands, highlighting the metabolic significance of BA synthesis rhythms.^10^^,^^15^

Fatty acid translocase (CD36) is a versatile class B scavenger receptor that plays a crucial role in lipid homeostasis and immune responses.^16^ Increased CD36 levels in the liver could lead to an increase in key enzymes involved in BA synthesis, such as CYP7A1 and CYP27A1.^17^ In addition, CD36 regulates mouse gallstone susceptibility by altering biliary lipid content and gallbladder contractility, thereby impacting BA metabolism and secretion.^18^ These studies suggest that CD36 may have multiple regulatory effects on BAs. Our research group previously identified CD36 as a key regulator of the mammalian circadian clock, which plays an essential role in synchronizing the liver clock with glucose homeostasis.^19^ In subsequent screenings, we revealed that CD36 was associated with the circadian rhythm of BA metabolism. Currently, there is a notable lack of relevant research on the rhythmic regulatory function of CD36 in BA metabolism, PBC, and PSC. These unknown areas require further investigation.

In this study, we employed a bile duct ligation (BDL) model of cholestasis in both CD36 wild-type and liver-specific knockout mice to elucidate the role of CD36 in BA metabolism and the circadian rhythm. Our findings revealed that cholestasis disrupted the liver clock and BA metabolism rhythms, with abnormal and excessive diurnal expression of CD36. Importantly, CD36 overexpression specifically disrupted the rhythmic expression of 3-hydroxy-3-methylglutaryl-coenzyme A reductase (HMGCR) and CYP7A1, key enzymes involved in BA synthesis. Inhibition of CD36 expression normalized BA levels by restoring the rhythmicity of the HMGCR/CYP7A1 axis, thereby alleviating cholestatic liver injury and inflammation. These results highlight the role of CD36 in regulating BA metabolic rhythms and underscore its potential as a therapeutic target for cholestatic liver diseases by modulating the HMGCR/CYP7A1 axis.

## Materials and methods

### Human PBC and PSC samples

Liver biopsy samples were obtained from patients with PSC (*n* = 6) and PBC (*n* = 5). Control liver tissues were obtained from noncancerous areas adjacent to primary liver cancer tissues. All PBC and PSC patients included in this study were newly diagnosed and had not received any prior medical treatment. For the classification of PSC patients, the disease stages were defined as follows: stage 1 is characterized by inflammation and connective tissue proliferation surrounding the small bile ducts; stage 2 is characterized by the extension of inflammation into the liver parenchyma with the formation of scar tissue; stage 3 involves more severe fibrosis; and stage 4 corresponds to biliary cirrhosis. Stages 1 and 2 are considered early-stage PSCs, whereas stages 3 and 4 represent late-stage to advanced-stage PSCs, which share similar pathological features. For PBC patients, the diagnosis was established based on the presence of at least two of the following criteria: biochemical evidence of cholestasis, positive antimitochondrial antibody, or histological findings of florid duct lesions. All participants provided written informed consent, and the Ethics Committee of the Second Affiliated Hospital of Chongqing Medical University approved this study. The details regarding the demographic and clinical factors, including sex, age, and histological diagnosis, are shown in Table S1.

### Animals

Male C57BL/6J mice (Vital River Laboratories, Beijing, China) were used in this study. For the BDL model, 10-week-old male mice underwent sham surgery or bile duct ligation (BDL) for 1 week to induce cholestatic liver injury, and their effects on liver injury and BA metabolism were investigated. In another cohort, CD36^fl/fl^ mice (engineered with LoxP sites surrounding exon 5 of CD36) were crossed with albumin-Cre transgenic mice to generate liver-specific CD36 knockout (CD36 LKO) mice, as described previously.^19^ Liver samples were collected every 4 h over a 24 h period to assess circadian rhythms in BA metabolism and gene expression. For targeted CD36 inhibition, MC3 lipid nanoparticles loaded with CD36-siRNA (0.4 μg/g body weight) were intravenously injected into each mouse at ZT10, and the mice were sacrificed at ZT16 on day 7 to evaluate the therapeutic potential of CD36 inhibition during peak expression. Additionally, the mice in the three groups (SHAM, BDL, and BDL + CD36 LKO) were euthanized at ZT0 and ZT12 on day 7 to examine the effects of CD36 knockout on cholestatic liver injury and BA metabolism. All the mice were housed in a specific pathogen-free environment under a 12 h/12 h light/dark cycle with free access to standard chow and sterile water. Zeitgeber time 0 (ZT0) was defined as 6:00 a.m. All procedures complied with the guidelines of the Institutional Animal Care and Use Committee of Chongqing Medical University.

### Cell culture

HepG2 (ATCC, USA) and AML12 cells were cultured in Dulbecco's modified Eagle medium (HyClone, USA) supplemented with 10% fetal bovine serum and 100 U/mL penicillin‒streptomycin at 37 °C in a humidified incubator containing 5% CO_2_. All the cell lines were confirmed to be free of mycoplasma contamination. HepG2 and AML12 cells were treated with 200 μM cholic acid, chenodeoxycholic acid, or deoxycholic acid for 6 h.

### Lipid nanoparticle preparation for siRNA delivery

siRNA was dissolved in 2 mM citrate buffer (pH 3.5). Lipids at the desired molar ratio were dissolved in ethanol. The molar ratio of the constituent lipids was 55% Dlin-MC3-DMA (MC3), 10% Helper lipid (DSPC), 33.5% cholesterol, and 1.5% DMG-PEG (1,2-dimyristoyl-sn-glycerol, methoxypolyethylene glycol, PEG chain molecular weight: 2000). The lipid solution was then combined with the siRNA solution via a nanoassemblr microfluidic device (Precision NanoSystems, Vancouver, BC, Canada) at a flow rate ratio of 1:3 ethanol:aqueous phases. The mixed material was then diluted with a 3 × volume of 10 mM Tris buffer (pH 7.4) containing 9% sucrose, reducing the ethanol content to 6.25%. The diluted formulation was then concentrated by tangential flow filtration using dialysis tubing (30 kDa MWCO), followed by diafiltration against 10 volumes of 10 mM Tris buffer (pH 7.4) containing 9% sucrose. After diafiltration, the formulations were then concentrated to the desired siRNA concentration, followed by filling into vials and freezing. The siRNA sequences used were as follows: CD36 siRNA: GCUAUUGCGACAUGAUUAAUGTT, CAUUAAUCAUGUCGCAAUAGCTT; control siRNA: UUCUCCGAACGUGUCACGUTT, ACGUGACACGUUCGGAGAATT.

### RNA extraction and quantitative real-time PCR

Total RNA was extracted from both cellular samples and various mouse tissues via the TRIzol reagent (TaKaRa, Japan). Following this extraction process, the RNA was reverse-transcribed into complementary DNA via the PrimeScript RT Kit (TaKaRa, Japan) in accordance with the specific protocols outlined in the manufacturer's instructions. Subsequently, quantitative reverse transcription PCR was performed via the Bio-Rad CFX Connect system, a reliable platform manufactured by Bio-Rad, USA. SYBR Green PCR Mix (TaKaRa, Japan) was used for amplification. To ensure the accuracy of the gene expression assessment, the observed expression levels were normalized to those of β-actin. Relative gene expression was quantified via the well-established 2^−ΔΔCt^ method, which allows for comparative analysis between sample groups. The sequences of primers used in these experiments are listed in Tables S2 and S3.

### Western blotting

Protein extracts from liver tissues were prepared via radioimmunoprecipitation assay (RIPA) buffer, separated via SDS‒PAGE, and transferred onto PVDF membranes. The membranes were subsequently incubated with primary antibodies at 4 °C overnight. The membranes were subsequently washed three times with Tris-buffered saline with Tween 20 (TBST; 10 min each) before they were incubated with horseradish peroxidase-conjugated secondary antibodies at room temperature for 1 h. The membranes were then washed three times with TBST for 15 min each. Protein detection was performed via an enhanced chemiluminescence kit (Amersham Bioscience, US), and quantitative analysis was conducted via ImageJ software (v1.53k). The primary antibodies utilized included anti-CD36 (Novus, Cat# NB400-144, RRID:AB_10003498), anti-HMGCR (ABclonal, Cat# A19063, RRID:AB_2862556), anti-CYP7A1 (Affinity Biosciences, Cat# DF2612, RRID:AB_2839818), anti-NR0B2 (Affinity Biosciences, Cat# DF6648, RRID:AB_2838610), anti-AQP8 (ABclonal, Cat# A8539, RRID:AB_2768409), and anti-β-actin (Proteintech, Cat# 20536-1-AP, RRID:AB_10700003) antibodies.

### **Histology**

Tissue sections (5 μm thickness) were paraffin-embedded and processed for hematoxylin and eosin (H&E) staining and Sirius red staining.

### Immunohistochemistry

Liver tissue sections were fixed in 4% paraformaldehyde and embedded in paraffin for immunohistochemical analysis. To quench endogenous peroxidase activity, the sections were treated with 3% H_2_O_2_, followed by blocking with goat serum. Primary antibody incubation was performed at 4 °C overnight with F4/80 (1:800; CST, Cat# 30325, RRID:AB_2798990) and CK19 (1:1000; Servicebio, Cat# GB11197-100) antibodies. The sections were then washed and incubated with secondary antibodies at 37 °C for 30 min. Immunoreactivity was visualized via a diaminobenzidine kit, and the sections were counterstained with hematoxylin. Microscopic images were captured via a Zeiss microscope.

### Biochemical analysis

Serum and liver samples were collected from anesthetized mice. The serum levels of alkaline phosphatase (ALP; Cat# A059-2), gamma-glutamyl transferase (GGT; Cat# C017-2-1), aspartate aminotransferase (AST; Cat# C010-2-1), alanine aminotransferase (ALT; Cat# C009-2-1), and total BAs (TBA; Cat# E003-2-1) were measured via enzymatic kits (Nanjing Jiancheng, China). The concentration of 7α-hydroxy-4-cholesten-3-one (C4) was determined via an ELISA kit (MEI KE). Total cholesterol (TC; Nanjing Jiancheng, Cat# F002-1-1), free cholesterol (FC; AIDISHENG, Cat# ADS-W-ZF015), nonesterified fatty acids (NEFA; Nanjing Jiancheng, Cat# A042-2-1), phospholipids (PL; AIDISHENG, Cat# ADS-W-D034), and TBA were measured enzymatically, and cholesterol esters (CE) were quantified via ELISA kits (MEI KE).

### Statistical analysis

Statistical analyses were performed via GraphPad Prism version 9.5. The data were presented as mean ± SEM. Group comparisons were performed via two-tailed unpaired Student's *t*-test or two-way ANOVA. A *p*-value less than 0.05 was considered statistically significant. Rhythmic analysis was conducted via the Circacompare package in R with a period of 24 h. For genes showing rhythmicity in two or more groups, the amplitude and phase were compared. The significance threshold for rhythm detection was set at *p* < 0.05, with an amplitude filtering criterion of > 10% of the total expression level. Intergroup differences in amplitude (ΔAMP) and phase (ΔPhase) were assessed via likelihood ratio tests with a significance threshold of *p* < 0.05. Mesor stability (baseline expression level) was validated through model residual analysis.

## Results

### BA metabolism rhythm and core clock gene oscillation were disrupted in cholestatic liver injury mice

**Figure 1:**
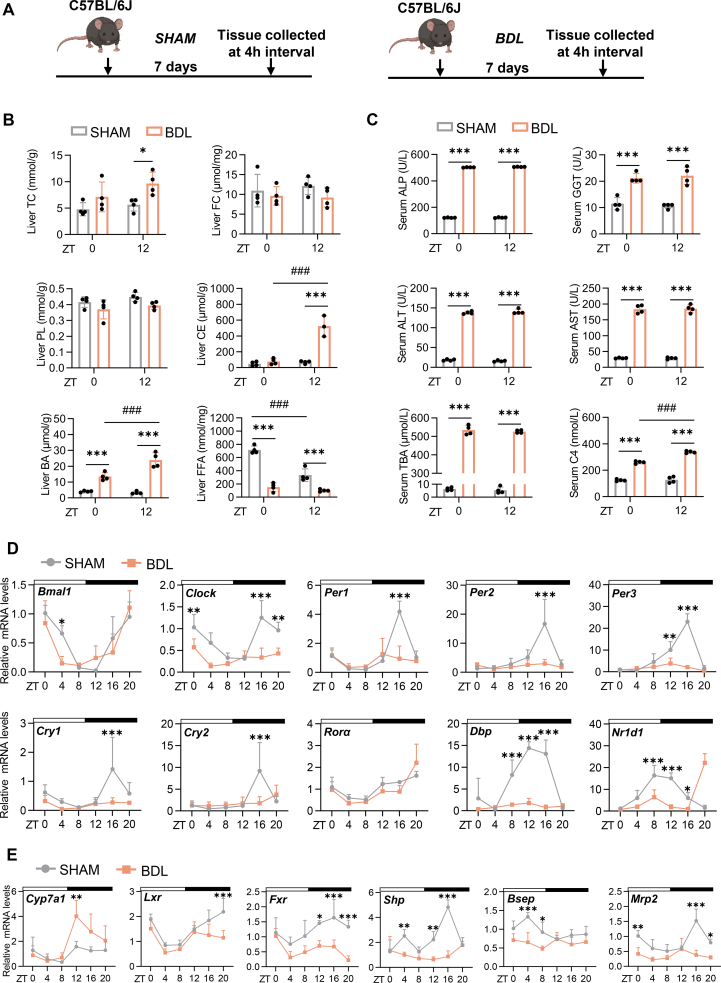
Bile acid metabolism rhythm and core clock gene oscillation were disrupted in mice with cholestatic liver injury. **(A)** Experimental approach for establishing the animal model. The figure was created via BioRender.com. **(B)** Hepatic levels of TC, FC, PL, CE, BA, and FFA in the SHAM and BDL mice at ZT0 and ZT12. **(C)** Serum levels of ALP, GGT, ALT, AST, TBA, and C4 in the SHAM and BDL mice at ZT0 and ZT12. **(D)** mRNA expression levels of core clock genes in the livers of SHAM and BDL mice over a 24-h period. **(E)** mRNA expression levels of key genes involved in bile acid synthesis, regulation, and secretion in the livers of SHAM and BDL mice over 24 h (*n* = 4 per time point per group). The data were shown as mean ± SEM. Group comparisons were performed via two-way ANOVA. ∗*p* < 0.05, ∗∗*p* < 0.01, and ∗∗∗*p* < 0.001 versus the control groups; ^#^*p* < 0.05, ^##^*p* < 0.01, and ^###^*p* < 0.001 for ZT0 versus ZT12 within the same group. ZT0 refers to the beginning of the subjective circadian period (6:00 a.m.). The black bar represents the dark phase from 6:00 p.m. to 6:00 a.m. TC, total cholesterol; FC, free cholesterol; PL, phospholipid; BA, bile acid; CE, cholesteryl ester; FFA, free fatty acid; BDL, bile duct ligation; ALP, alkaline phosphatase; ALT, alanine aminotransferase; AST, aspartate aminotransferase; GGT, gamma-glutamyl transferase; C4, 7α-hydroxy-4-cholesten-3-one; TBA, total bile acids.

**Fig. S1:**
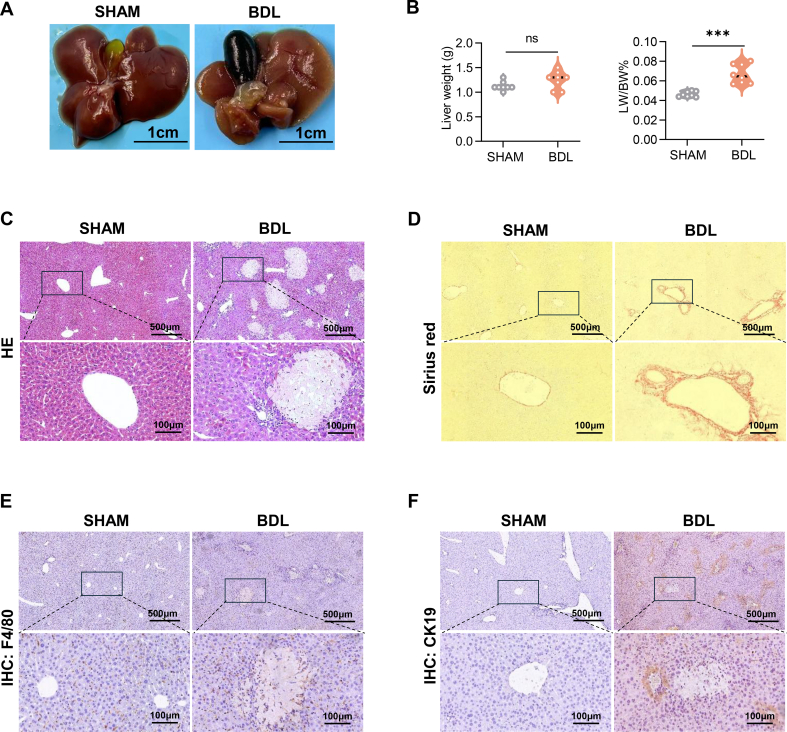
A cholestatic liver injury mouse model was successfully established. **(A)** Representative images of liver and gallbladder tissues from the SHAM and BDL mice at ZT0 and ZT12. Scale bar: 1 cm. **(B)** Liver weights and liver weight-to-body weight ratios of the different groups. **(C)** Representative images of hematoxylin-eosin-stained liver tissue from the SHAM and BDL mice. **(D)** Representative images of liver tissue from the SHAM and BDL mice stained with Sirius red. **(E)** Representative images of liver tissue from the SHAM and BDL mice subjected to immunohistochemistry staining for F4/80. **(F)** Representative images of liver tissue from the SHAM and BDL mice subjected to immunohistochemistry staining for CK19. Scale bars: 100 or 500 μm. *n* = 4 per time point per group. The data were shown as mean ± SEM. Group comparisons were performed via *t*-tests. ∗*P* < 0.05, ∗∗*P* < 0.01, and ∗∗∗*P* < 0.001 versus the control group; ns, not significant. ZT0 refers to the start of the subjective circadian period (6:00 a.m.). CD36, cluster of differentiation 36; BDL, bile duct ligation.

Although some studies have indicated a reciprocal influence between BAs and circadian rhythms, research specifically addressing circadian rhythms in cholestatic liver disease remains limited.^20^, ^21^, ^22^, ^23^ To investigate the role of the circadian rhythm in cholestatic liver disease, we first established a model using sham-operated (SHAM) and bile duct ligation (BDL) mice, which is a standard approach for inducing cholestatic liver fibrosis. We collected liver tissue from both SHAM and BDL mice every 4 h over a 24 h period (Fig. 1A). Phenotypic verification demonstrated that the BDL mice exhibited significant liver injury and fibrosis, as evidenced by various histopathological changes (Fig. S1A–S1F). Diurnal analysis of hepatic lipid content revealed no significant differences in TC, free FC, or PL levels between SHAM and BDL mice (Fig. 1B). However, CE level increased at ZT12, and BA level increased at both ZT0 and ZT12, whereas FFA level decreased at these time points in the BDL mice. Indeed, day‒night variations in CE and BA levels, but not in FFA levels, were observed in the BDL mice (Fig. 1B). Serum analysis at ZT0 and ZT12 revealed elevated levels of ALP, GGT, ALT, AST, C4, and TBA in the BDL group. Notably, C4 levels exhibited diurnal variation, suggesting disrupted BA synthesis rhythms in BDL mice (Fig. 1C).

The core circadian clock mechanism involves transcription‒translation feedback loops regulated by Bmal1 and Clock, which activate genes such as period (Per1/Per2/Per3) and cryptochrome (Cry1/Cry2) and are further modulated by Rev-erbs and Rors.^24^ To assess the effect of cholestatic liver disease on circadian gene expression, we analyzed the 24 h mRNA profiles of core clock genes. Compared with those in the SHAM mice, most clock genes in the BDL mice presented reduced amplitudes, and the rhythmic expression of Per1, Per2, and nuclear receptor subfamily 1 group D member 1 (Nr1d1) was absent (Fig. 1D; Table S4). Furthermore, we investigated BA metabolism genes and found that the amplitude of Cyp7a1 increased while that of liver X receptor (Lxr) decreased in the BDL mice and that the rhythmic expression of Fxr, bile salt export pump (Bsep), and multidrug resistance protein 2 (Mrp2) was also abolished (Fig. 1E; Table S4). Overall, these results indicate that the gene oscillation patterns of BA metabolism rhythms and the core circadian clock are significantly influenced by cholestatic liver injury.

### Hepatic CD36 **regulated** the circadian rhythm of genes involved in BA synthesis and the core clock

**Figure 2:**
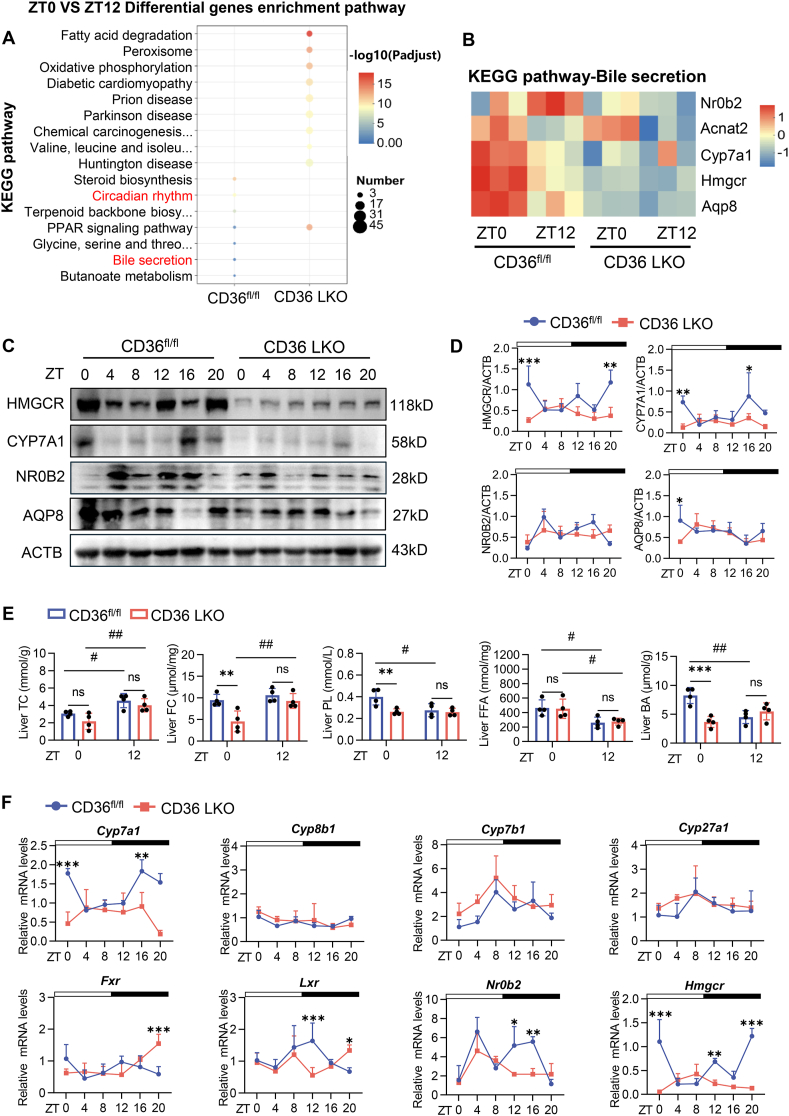
Hepatic CD36 regulated the circadian rhythm of genes involved in bile acid synthesis and the core clock. **(A)** KEGG pathway enrichment analysis of ZT0 versus ZT12 differential genes between CD36^fl/fl^ and CD36 LKO mice. The *y*-axis shows the enriched KEGG pathways, and the *x*-axis represents the groups. The dot size indicates the number of genes involved in each pathway, and the color reflects the adjusted *p*-value significance (*P*_adj_). **(B)** Heatmap of the expression levels of bile acid metabolism-related rhythmic genes in the CD36^fl/fl^ and CD36 LKO mice at ZT0 and ZT12. **(C)** Diurnal expression levels of the HMGCR, CYP7A1, NR0B2, and AQP8 proteins in the liver tissues of the CD36^fl/fl^ and CD36 LKO mice, as determined by Western blotting analyses. **(D)** Relative quantification of diurnal HMGCR, CYP7A1, NR0B2, and AQP8 protein expression in liver tissues from CD36^fl/fl^ and CD36 LKO mice. **(E)** Hepatic TC, FC, PL, FFA, and BA levels in CD36^fl/fl^ and CD36 LKO mice at ZT0 and ZT12. **(F)** mRNA levels of key genes involved in bile acid synthesis, regulation, and cholesterol synthesis were detected over 24 h in the livers of the CD36^fl/fl^ and CD36 LKO mice. *n* = 3–4 per time point per group. The data were presented as mean ± SEM. Group comparisons were performed via two-way ANOVA. ∗*p* < 0.05, ∗∗*p* < 0.01, and ∗∗∗*p* < 0.001 versus the control groups; ^#^*p* < 0.05, ^##^*p* < 0.01, and ^###^*p* < 0.001 for ZT0 versus ZT12 within the same group; ns, not significant. ZT0 refers to the beginning of the subjective circadian period (6:00 a.m.). The black bar represents the lights-off period from 6:00 p.m. to 6:00 a.m. HMGCR, 3-hydroxy-3-methylglutaryl-coenzyme A reductase; CYP7A1, cholesterol 7α-hydroxylase; NR0B2, nuclear receptor subfamily 0 group B member 2; AQP8, aquaporin 8; TC, total cholesterol; FC, free cholesterol; PL, phospholipid; BA, bile acid; FFA, free fatty acid.

**Fig. S2:**
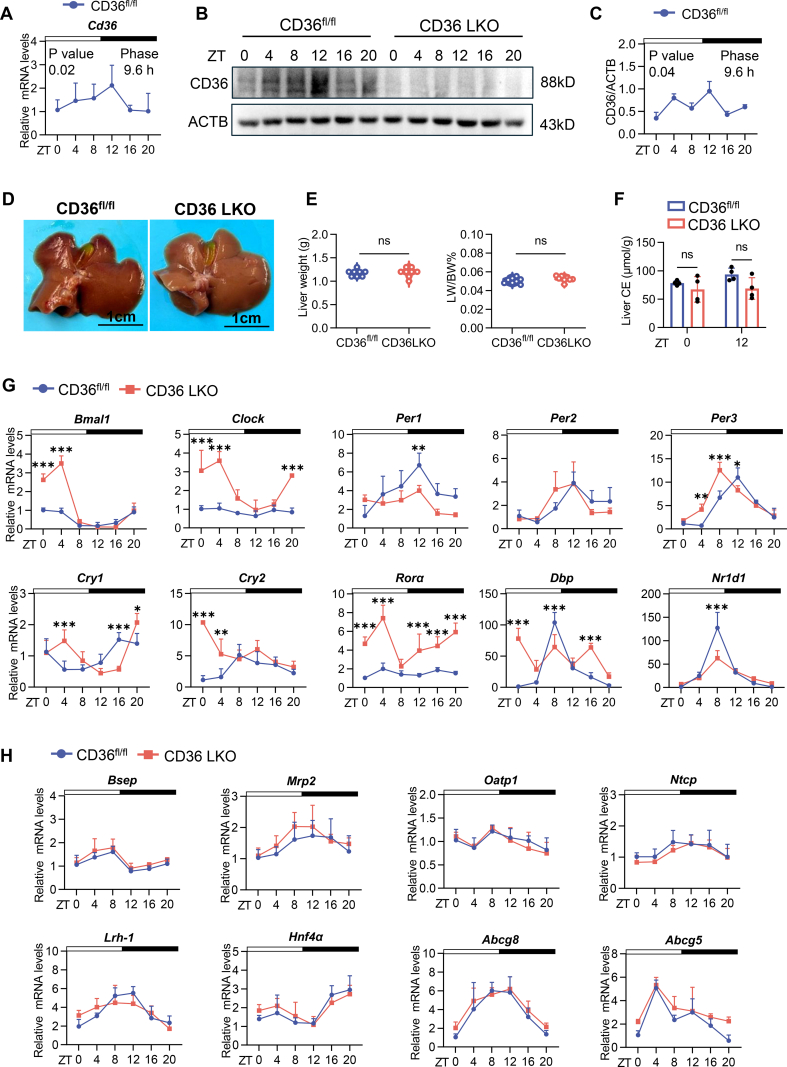
Hepatocyte CD36 plays an important role in maintaining the core clock gene rhythm. **(A)** mRNA expression levels of CD36 in the livers of the CD36^fl/fl^ mice over a 24-h period. **(B)** Diurnal CD36 protein expression levels from western blotting analysis of liver tissues from the CD36^fl/fl^ and CD36 LKO mice. **(C)** Relative quantification of CD36 protein diurnal expression in liver tissues from the CD36^fl/fl^ mice. **(D)** Representative images of liver and gallbladder tissues from the CD36^fl/fl^ and CD36 LKO mice at ZT0 and ZT12. Scale bar: 1 cm. **(E)** Liver weights and liver weight-to-body weight ratios of the different groups. **(F)** Hepatic CE levels in the CD36^fl/fl^ and CD36LKO mice at ZT0 and ZT12. **(G)** mRNA expression levels of core clock genes in the livers of the CD36^fl/fl^ and CD36LKO mice over a 24 h period. **(H)** mRNA expression levels of key genes involved in bile acid secretion, reabsorption, regulation, and cholesterol efflux in the livers of the CD36^fl/fl^ and CD36LKO mice over 24 h *n* = 3–4 per time point per group. The data were presented as mean ± SEM. Group comparisons were performed via two-way ANOVA. ∗*P* < 0.05, ∗∗*P* < 0.01, and ∗∗∗*P* < 0.001 versus the control group. ns, not significant. ZT0 refers to the beginning of the subjective circadian period (6:00 a.m.). The black bar indicates the dark phase from 6:00 p.m. to 6:00 a.m. CD36, cluster of differentiation 36; CE, cholesteryl ester.

CD36, a key protein involved in lipid and glucose metabolism, has been implicated in BA synthesis and reabsorption.^25^^,^^26^ Notably, our previous research demonstrated that CD36 plays an essential role in regulating the circadian rhythm.^19^ Given the observed disruption of BA rhythmicity in BDL mice, we hypothesized that CD36 may introduce a novel crosstalk between the circadian rhythm and BA metabolism, potentially revealing a distinct mechanism. CD36 exhibited clear diurnal variation in mRNA and protein levels, peaking at night (Fig. S2A–S2C), which was consistent with previous findings.^19^ KEGG analysis of the RNA sequencing data revealed significant enrichment of diurnally differential genes within the terms “circadian rhythm” and “bile secretion” (Fig. 2A), and the heatmap illustrated diurnal variations in key genes that were blunted by CD36 LKO, particularly in BA synthesis genes, including Hmgcr, Cyp7a1, and the negative feedback regulator nuclear receptor subfamily 0 group B member 2 (Nr0b2) (Fig. 2B). Consistently, the protein levels of HMGCR, CYP7A1, NR0B2, and aquaporin 8 (AQP8) displayed distinct diurnal variations in the CD36^fl/fl^ mice but not in the CD36 LKO mice (Fig. 2C and D), suggesting that CD36 maintains the rhythmic amplitude of BA synthesis genes.

There were no notable differences in liver morphology or the liver weight/body weight ratio between the CD36^fl/fl^ and CD36 LKO mice (Fig. S2D and S2E). However, we observed a significant reduction in FC, PL, and BA levels, whereas no significant differences in TC, FFA, and CE contents were observed at ZT0. Importantly, diurnal variations in the PL and BA levels were eliminated in the CD36 LKO mice (Fig. 2E; Fig. S2F). To investigate the role of CD36 in the regulation of the circadian clock, we analyzed the 24 h mRNA profiles of core clock genes. The rhythmic expression of D-site-binding protein (Dbp) was diminished in the CD36 LKO mice, and the phases and amplitudes of most genes also changed (Table S5; Fig. S2G). We also examined BA metabolism genes and found that the rhythmic expression of Cyp27a1 and Lxr was abolished in the CD36 LKO mice, whereas the phase or amplitude of Cyp7a1, Cyp8b1, and Hmgcr was significantly altered (Fig. 2F; Fig. S2H; Table S5). Taken together, these results indicate that hepatic CD36 plays a pivotal role in regulating the circadian rhythm of genes involved in the core clock and BA synthesis.

### Hepatic CD36 was elevated in patients with PBC and PSC and displayed abnormally robust diurnal expression in mice with cholestatic liver injury

**Figure 3:**
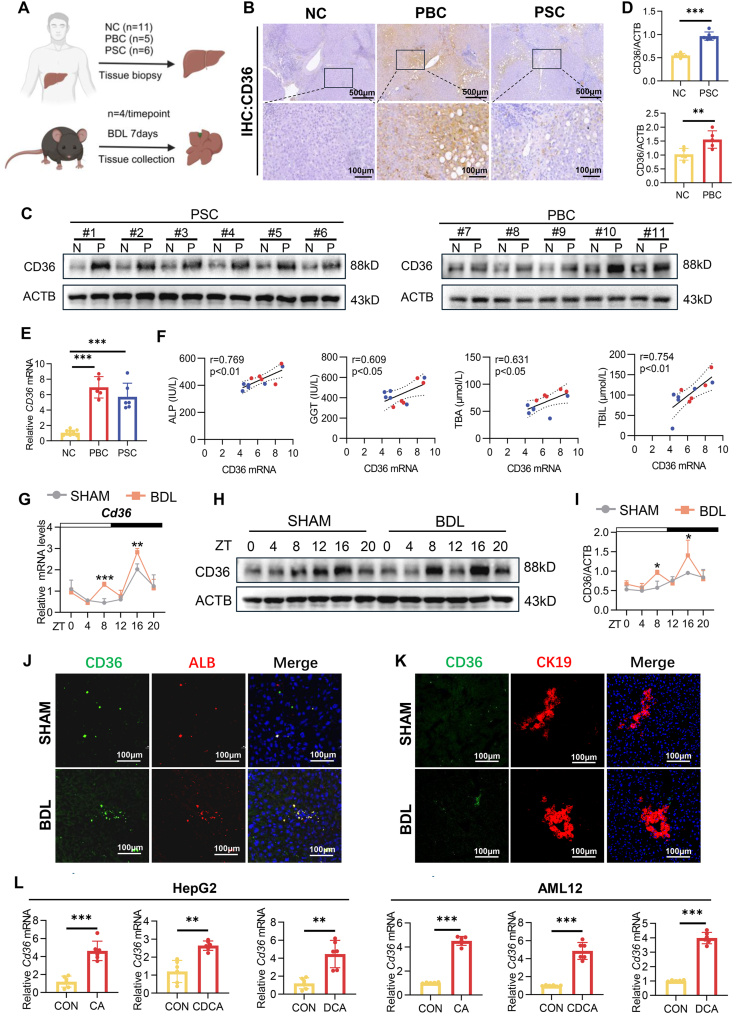
Hepatic CD36 was elevated in patients with PBC and PSC, and CD36 displayed abnormally robust diurnal expression in mice with cholestatic liver injury. **(A)** Schematic representation of the study design for the clinical and animal experiments. The figure was created via BioRender.com. **(B)** Representative images of liver tissue subjected to immunohistochemistry staining (IHC) for CD36 in normal controls (NCs), PBC patients, and PSC patients. Scale bars: 100 or 500 μm. **(C)** Western blotting analysis of CD36 expression in the livers of NC (*n* = 11), PBC (*n* = 5), and PSC (*n* = 6) patients. **(D)** Relative quantification of CD36 protein expression in livers from NC (*n* = 11), PBC (*n* = 5), and PSC (*n* = 6) patients. **(E)** mRNA expression levels of CD36 in the livers from NC (*n* = 11), PBC (*n* = 5), and PSC (*n* = 6) patients. **(F)** Linear regression analysis of the correlations between hepatic CD36 mRNA expression and serum ALP, GGT, TBA, and TBIL levels. **(G)** mRNA expression levels of CD36 in the livers of SHAM and BDL mice (*n* = 4 per time point per group) over a 24 h period. **(H)** Diurnal CD36 protein expression levels from Western blotting analysis of liver tissues from the SHAM and BDL mice (*n* = 3 per time point per group). **(I)** Relative quantification of CD36 protein diurnal expression in liver tissues from the SHAM and BDL mice. **(J)** Double immunofluorescence staining for CD36 (green) and ALB (red) in the SHAM and BDL mice. Nuclei were counterstained with DAPI (blue). Scale bar: 100 μm. **(K)** Double immunofluorescence staining for CD36 (green) and CK19 (red) in the SHAM and BDL mice. Nuclei were counterstained with DAPI (blue). Scale bar: 100 μm. **(L)** Quantitative reverse transcription PCR analysis of CD36 mRNA levels in HepG2 and AML12 cells cultured for 6 h with cholic acid (CA), chenodeoxycholic acid (CDCA), and deoxycholic acid (DCA) at the indicated doses (*n* = 6). All the data were presented as mean ± SEM. Group comparisons were performed via two-way ANOVA. ∗*p* < 0.05, ∗∗*p* < 0.01, and ∗∗∗*p* < 0.001 versus the control group. ZT0 refers to the beginning of the subjective circadian period (6:00 a.m.). The black bars indicate the dark phase from 6:00 p.m. to 6:00 a.m. PBC, primary biliary cholangitis; PSC, primary sclerosing cholangitis; ALP, alkaline phosphatase; GGT, gamma-glutamyl transferase; TBA, total bile acids; TBIL, total bilirubin; BDL, bile duct ligation; ALB, albumin; CD36, cluster of differentiation 36.

To assess CD36 expression in cholestatic liver disease, we analyzed its level in liver samples from patients with PBC and PSC, as well as in cholestatic mice (Fig. 3A). Immunohistochemistry analysis revealed increased CD36 expression in the liver tissues of patients with PBC and PSC (Fig. 3B), with higher protein levels than those of normal controls (Fig. 3C and D). Consistently, CD36 mRNA expression was elevated in patients with PBC and PSC (Fig. 3E). Pearson's correlation analysis revealed positive associations between serum markers (ALP, GGT, TBA, and TBIL) and liver CD36 levels (Fig. 3F). In the BDL mice, CD36 mRNA and protein levels were increased at ZT8 and ZT16, respectively (Fig. 3G–I). Dual immunofluorescence staining of albumin (ALB) and CD36, as well as CK19 and CD36, demonstrated that CD36 was predominantly expressed in hepatocytes rather than in bile duct cells. Notably, the expression of CD36 in hepatocytes increased following BDL (Fig. 3J and K). Additionally, *in vitro* experiments revealed that treatment of HepG2 cells with various BAs (cholic acid, chenodeoxycholic acid, and deoxycholic acid) significantly up-regulated CD36 expression, a finding that was also observed in mouse hepatocyte AML12 cells (Fig. 3L). Collectively, these results indicate that CD36 expression is markedly increased under cholestatic conditions.

### CD36 LKO attenuated cholestatic liver injury by restoring the diurnal difference in BA

**Figure 4:**
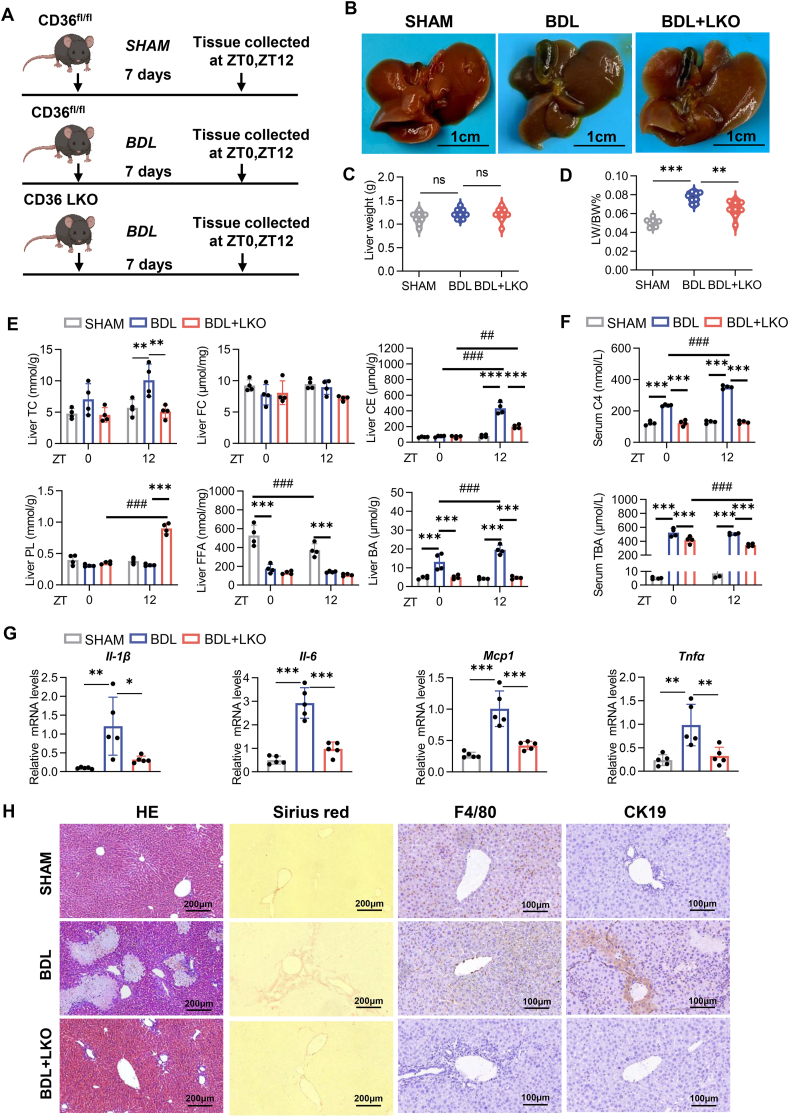
CD36 LKO attenuated cholestatic liver injury by restoring the diurnal difference in bile acid. **(A)** Experimental approach for establishing the animal model. The figure was created via BioRender.com. **(B)** Representative images of liver and gallbladder tissues from the SHAM, BDL, and BDL + LKO mice at ZT0 and ZT12. Scale bar: 1 cm. **(C)** Liver weights of the different groups. **(D)** Liver weight-to-body weight ratios of the different groups. **(E)** Hepatic levels of TC, FC, CE, PL, FFA, and BA in the SHAM, BDL and BDL + LKO mice at ZT0 and ZT12. **(F)** Serum levels of C4 and TBA in the SHAM, BDL and BDL + LKO mice at ZT0 and ZT12. **(G)** Hepatic mRNA levels of the indicated genes. **(H)** Representative images of liver tissue from SHAM, BDL and BDL + LKO mice subjected to hematoxylin-eosin, Sirius red, and immunohistochemistry staining for F4/80 and CK19. Scale bars: 100 or 200 μm. *n* = 4 per time point per group. The data were presented as mean ± SEM. Group comparisons were conducted via two-way ANOVA. ∗*p* < 0.05, ∗∗*p* < 0.01, and ∗∗∗*p* < 0.001 versus the control group; ns, not significant. ZT0 marks the beginning of the subjective circadian period (6:00 a.m.). CD36, cluster of differentiation 36; BDL, bile duct ligation; TC, total cholesterol; FC, free cholesterol; PL, phospholipid; BA, bile acid; CE, cholesteryl ester; FFA, free fatty acid; C4, 7α-hydroxy-4-cholesten-3-one; TBA, total bile acids.

Next, we explored the role of CD36 in cholestatic liver injury via BDL surgery in CD36^fl/fl^ and CD36 LKO mice. Tissues and serum were collected at ZT0 and ZT12 after 7 days (Fig. 4A). Liver injury, as assessed by gross examination and the liver weight/body weight ratio, was alleviated in the CD36 LKO mice compared with the BDL mice (Fig. 4B–D). There were no significant differences in FC levels among the experimental groups, whereas BDL resulted in decreased FFA levels at both ZT0 and ZT12. TC levels increased in the BDL mice but decreased in the CD36 LKO mice at ZT12, and PL levels increased at ZT12 in the CD36 LKO mice. Notably, at ZT12, the levels of CE and BA were elevated in the BDL mice, whereas a decrease was observed in the CD36 LKO mice. Importantly, the pronounced diurnal induction of CE and BA caused by BDL was restored in the CD36 LKO mice (Fig. 4E).

Serum analysis revealed that the levels of C4 and TBA increased in the BDL mice but decreased in the CD36 LKO mice at both ZT0 and ZT12 (Fig. 4F). The expression of inflammatory genes, including IL-1β, IL-6, MCP-1, and TNFα, was significantly up-regulated in the BDL mice and down-regulated in the CD36 LKO mice (Fig. 4G). Histological analysis demonstrated that BDL-induced liver injury, collagen deposition, inflammatory infiltration, and cholangiocyte proliferation were significantly alleviated in the CD36 LKO mice (Fig. 4H). Collectively, these findings suggest that CD36 deficiency in hepatocytes mitigates cholestatic liver injury by restoring the diurnal differences in BA pools.

### CD36 LKO partially prevented **the** disturbances in the profile of the rhythmic transcriptome in the liver induced by BDL

**Figure 5:**
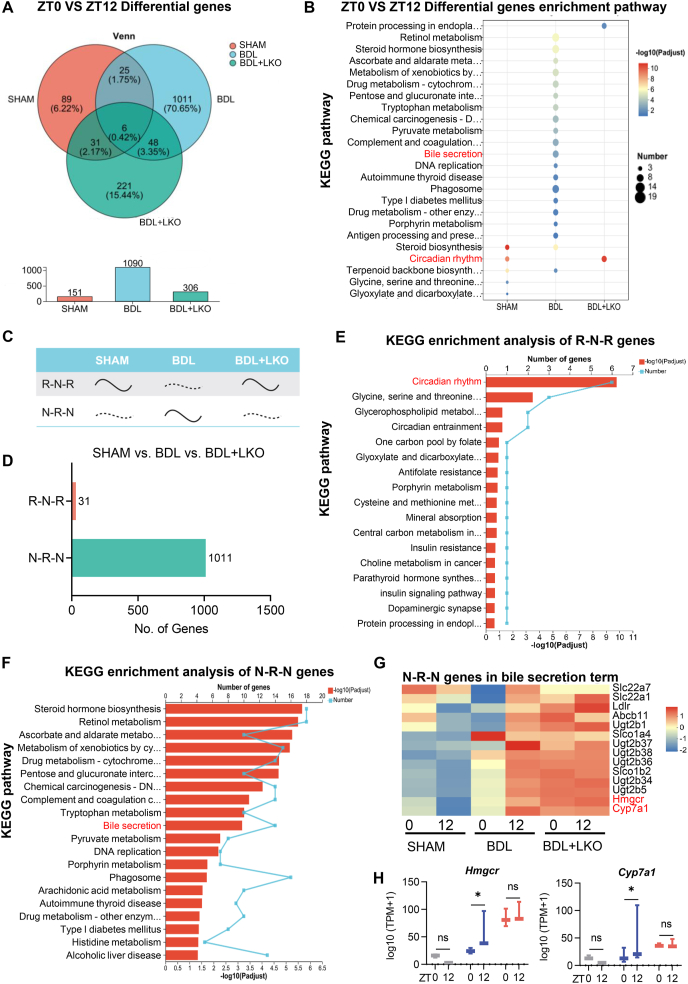
CD36 LKO partially prevented the disturbances in the profile of the rhythmic transcriptome in the liver induced by BDL. **(A)** Venn diagram of the number of rhythmic genes in the different groups. **(B)** KEGG pathway enrichment analysis of differentially expressed genes between ZT0 and ZT12 in different groups. **(C)** Schematic depiction of the two categories of alterations in the rhythmic transcriptome between the study groups considered. The dashed line indicates “nonrhythmic”. **(D)** The bar plot representing the number of transcripts present in each category. **(E)** KEGG pathway enrichment analysis of rhythmic genes whose rhythmic expression decreased after BDL but whose expression was reversed by CD36 LKO (R-N-R pattern). **(F)** KEGG pathway enrichment analysis of rhythmic genes whose rhythmic rhythm was abnormally increased by BDL but whose expression was reversed by CD36 LKO (N-R-N pattern). **(G)** Heatmap of the expression levels of genes with the N‒R‒N pattern in the “bile secretion” KEGG term in different groups at ZT0 and ZT12. **(H)** Expression levels of Hmgcr and Cyp7a1 in different groups. *n* = 3 per time point per group. Hmgcr, 3-hydroxy-3-methylglutaryl-coenzyme A reductase; Cyp7a1, cholesterol 7α-hydroxylase; CD36, cluster of differentiation 36; BDL, bile duct ligation.

To elucidate the specific molecular mechanism by which liver CD36 regulates the transcriptome of the liver circadian rhythm in BDL mice, we conducted diurnal transcriptome analysis and further clustering and enrichment analysis of oscillating genes. A total of 151, 1090, and 306 circadian rhythmic genes were identified in the SHAM, BDL, and BDL + LKO groups, respectively (Fig. 5A). Notably, these genes were significantly enriched in KEGG pathways related to “bile secretion” and “circadian rhythm” (Fig. 5B). Further examination of the circadian expression of genes altered by BDL and subsequently rescued by CD36 revealed two distinct patterns: R-N-R (rhythmic [R] in SHAM, non-rhythmic [N] in BDL, and rhythmic [R] in BDL + LKO) and N-R-N (non-rhythmic [N] in SHAM, rhythmic [R] in BDL, and non-rhythmic [N] in BDL + LKO) (Fig. 5C). Specifically, 2.17% of the genes (31 in total) conformed to the R‒N‒R pattern, whereas 70.65% of the genes (1101 in total) followed the N‒R‒N pattern (Fig. 5D). KEGG enrichment analysis indicated that genes within the R-N-R group were primarily associated with the “circadian rhythm” pathway, whereas genes in the N-R-N group were enriched predominantly in pathways related to “bile secretion” and “steroid hormone synthesis” (Fig. 5E and F). Heatmap analysis revealed that the N-R-N mode gene set enriched in the term “bile secretion” included several genes related to BA metabolism, of which Hmgcr and Cyp7a1 received particular attention (Fig. 5G). Transcriptome gene expression analysis revealed that BDL induced abnormal circadian variations in Hmgcr and Cyp7a1, which were reversed by CD36 LKO (Fig. 5H). CD36 deficiency influenced the circadian transcriptome in the livers of BDL mice in distinct rhythmic patterns, particularly affecting BA synthesis genes following the N‒R‒N pattern. These results underscore the crucial role of CD36 in the circadian rhythm of BA synthesis.

### CD36 reset abnormal diurnal differences in the core clock and BA synthesis of hepatocytes in BDL mice

**Figure 6:**
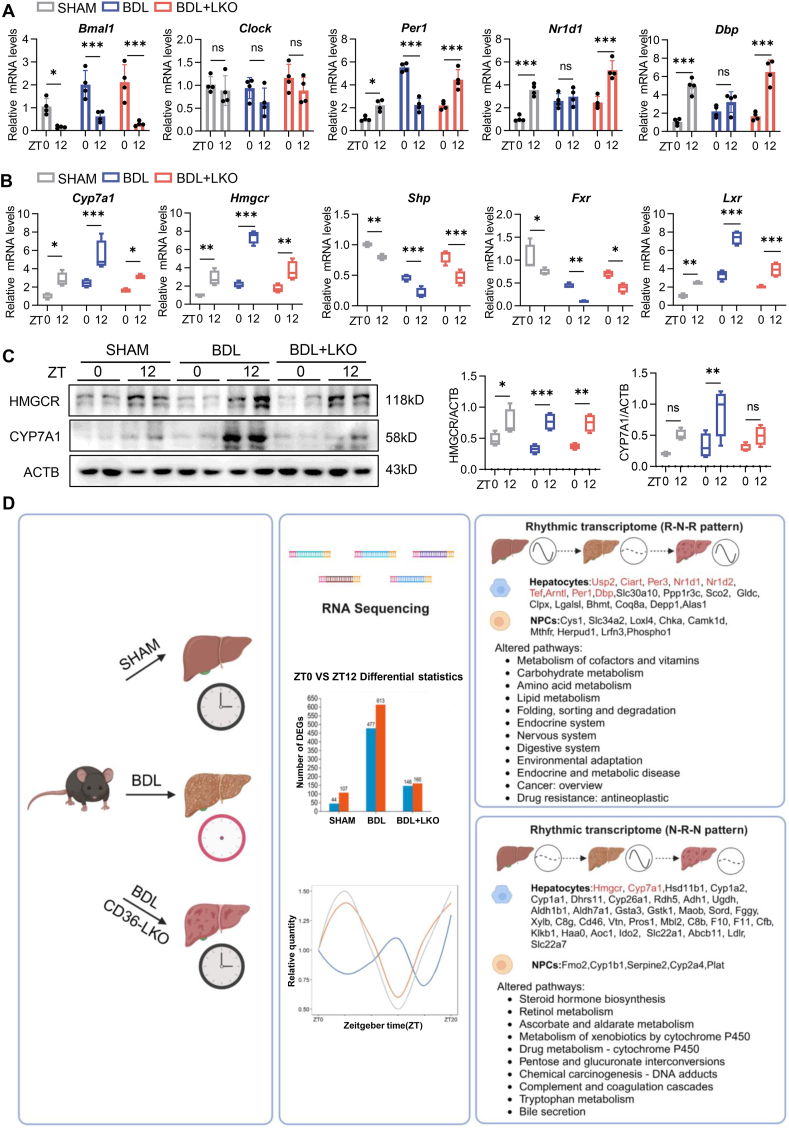
CD36 reset abnormal diurnal differences in the core clock and bile acid synthesis of hepatocytes in BDL mice. **(A)** mRNA expression levels of core clock genes in the livers of SHAM, BDL and BDL + LKO mice at ZT0 and ZT12. **(B)** mRNA expression levels of key genes involved in bile acid synthesis, regulation, and cholesterol synthesis in the livers of SHAM, BDL, and BDL + LKO mice at ZT0 and ZT12. **(C)** Protein expression levels of HMGCR and CYP7A1 in liver tissues of SHAM, BDL, and BDL + LKO mice at ZT0 and ZT12, as determined by Western blotting analyses. **(D)** Schematic representation of CD36-reprogrammed genes and pathways. *n* = 4 per time point per group. The data were presented as mean ± SEM. Group comparisons were conducted via two-way ANOVA. ∗*p* < 0.05, ∗∗*p* < 0.01, and ∗∗∗*p* < 0.001 versus the control group; ns, not significant. ZT0 marks the beginning of the subjective circadian period (6:00 a.m.). HMGCR, 3-hydroxy-3-methylglutaryl-coenzyme A reductase; CYP7A1, cholesterol 7α-hydroxylase; CD36, cluster of differentiation 36; BDL, bile duct ligation.

We validated the expression of genes related to circadian rhythm and BA metabolism via quantitative real-time PCR. In the BDL mice, the day‒night differences in Nr1d1 and Dbp mRNA expression were lost but were reversed in the CD36 LKO mice (Fig. 6A). Conversely, theenhanced circadian differences in Cyp7a1, Fxr, and Hmgcr mRNA expression observed in the BDL mice were diminished in the CD36 LKO mice (Fig. 6B). Similarly, the heightened diurnal variations in CYP7A1 and HMGCR protein levels detected in the BDL mice were abolished in the CD36 LKO mice (Fig. 6C). Based on our mouse model and RNA sequencing results, we summarized the circadian patterns of differential gene expression and the pathways affected by CD36 LKO. These selected genes were clustered into two categories: i) the R-N-R group (rhythmic [R] in SHAM, non-rhythmic [N] in BDL, and rhythmic [R] in BDL + LKO); and ii) the N-R-N group (non-rhythmic [N] in SHAM, gain rhythm [R] in BDL, non-rhythmic [N] in BDL + LKO) (Fig. 6D). The R-N-R group contained numerous metabolic and clock genes, whereas the N-R-N group included key genes involved in BA and cholesterol synthesis. From the perspective of cell expression categories, rhythmic genes regulated by CD36 are predominantly expressed in hepatocytes, with some expression observed in non-parenchymal cells (NPCs). These results suggest that CD36 reassembles the abnormal diurnal differencesof core clock genes and BA synthesis in the hepatocytes of BDL mice.

### CD36-siRNA delivered through lipid nanoparticles alleviated cholestatic liver injury in BDL mice by **improving** BA synthesis

**Figure 7:**
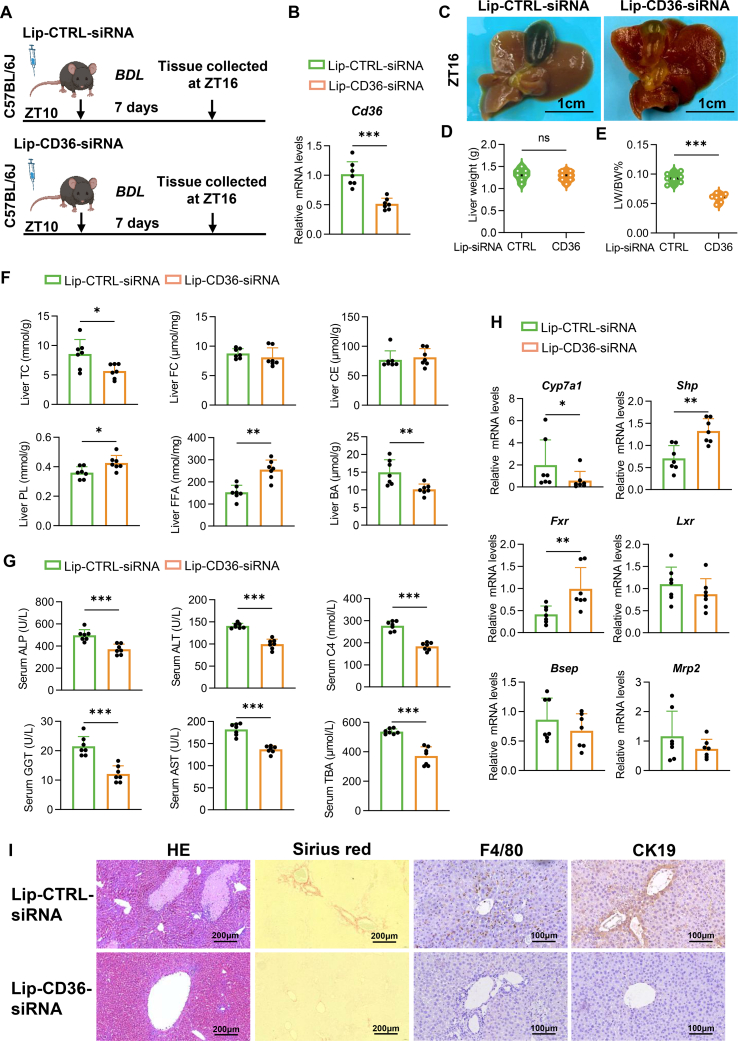
CD36-siRNA delivered through lipid nanoparticles at the diurnal peak alleviated cholestatic liver injury in·BDL mice by improving BA synthesis. **(A)** Experimental approach for establishing the animal model. The figure was created via BioRender.com. **(B)** mRNA expression levels of CD36 in the livers of the Lip-CTRL-siRNA and Lip-CD36-siRNA mice at ZT16. **(C)** Representative images of liver and gallbladder tissues from the Lip-CTRL-siRNA and Lip-CD36-siRNA mice at ZT16. Scale bar: 1 cm. **(D)** Liver weights of the different groups. **(E)** Liver weight-to-body weight ratios of the different groups. **(F)** Hepatic TC, FC, CE, PL, FFA, and BA levels in Lip-CTRL-siRNA and Lip-CD36-siRNA mice at ZT16. **(G)** Serum ALP, ALT, C4, GGT, AST, and TBA levels in the Lip-CTRL-siRNA and Lip-CD36-siRNA mice at ZT16. **(H)** mRNA expression levels of key genes involved in bile acid synthesis, regulation, and secretion in the livers of the Lip-CTRL-siRNA and Lip-CD36-siRNA mice at ZT16. **(I)** Representative images of liver tissue subjected to hematoxylin-eosin, Sirius red, and immunohistochemistry staining for F4/80 and CK19 from the Lip-CTRL-siRNA and Lip-CD36-siRNA mice. Scale bars: 100 or 200 μm. *n* = 7 per group. The data were presented as mean ± SEM. Group comparisons were conducted via two-way ANOVA. ∗*p* < 0.05, ∗∗*p* < 0.01, and ∗∗∗*p* < 0.001 versus the control group; ns, not significant. CD36, cluster of differentiation 36; BDL, bile duct ligation; TC, total cholesterol; FC, free cholesterol; PL, phospholipid; BA, bile acid; CE, cholesteryl ester; FFA, free fatty acid; C4, 7α-hydroxy-4-cholesten-3-one; TBA, total bile acids; ALP, alkaline phosphatase; ALT, alanine aminotransferase; AST, aspartate aminotransferase; GGT, gamma-glutamyl transferase.

Liposomal RNA nanocarriers have recently gained acceptance for the treatment of chronic liver disease and end-stage liver cancer owing to their targeted delivery and reduced toxicity.^27^ To explore circadian rhythm-based therapies, we administered lipid nanoparticles containing siCD36 at ZT10 to inhibit CD36 expression (Fig. 7A). This timing was strategically chosen to align with the peak expression of CD36, which occurs at ZT16 and corresponds to the maximum hepatocyte uptake of lipid nanoparticle-siRNA observed 6 h later in a previous study.^28^ Subsequent analysis of liver CD36 mRNA expression confirmed the effective knockdown of CD36 in the liver at ZT16 (Fig. 7B). Liver injury, as assessed by gross examination and the liver weight/body weight ratio, was alleviated following time-specific CD36 inhibition (Fig. 7C–E). Although there were no significant differences in the FC or CE content between the Lip-CTRL-siRNA-treated and Lip-CD36-siRNA-treated mice, the TC and BA levels were reduced, and the PL and FFA levels were increased in the CD36-inhibited mice (Fig. 7F). Serum analysis revealed improvements in key measures of liver damage and BA synthesis (Fig. 7G). Additionally, Cyp7a1 mRNA expression decreased, whereas Shp and Fxr levels increased in mice receiving CD36 inhibition (Fig. 7H). Histological analysis revealed that liver injury, collagen deposition, inflammation, and cholangiocyte proliferation were mitigated by CD36 inhibition (Fig. 7I). These results suggest that CD36 is integral to the rhythmic synthesis of BAs and that the delivery of CD36 inhibitors may be a potential therapeutic approach for cholestatic liver disease.

## Discussion

BA homeostasis is considered essential for the advancement of both the PBC and PSC.^29^ In this study, the diurnal oscillation of clock and BA metabolism genes was disrupted in mice subjected to BDL, and CD36 was up-regulated in the livers of patients with cholestasis and murine models. The inhibition of CD36 realigns the hepatic clock and BA metabolic cycle in mice, thus preventing the deterioration of cholestasis. These observations suggest that CD36 may be a clock target that regulates hepatic BA levels, thus contributing to the pathogenesis of cholestasis. The findings of this study underscore the vital function of CD36 as a mediator in cholestasis and indicate that targeting the circadian regulation of CD36 may represent a promising avenue for anticholestatic therapy.

BA homeostasis, which is primarily regulated by the liver, is crucial for preventing cholestatic liver disease caused by disruptions due to mutations in synthesis and transport genes.^11^ Approximately 10% of liver genes, including those encoding BA-metabolizing enzymes, exhibit rhythmic expression,^10^^,^^15^ and the absence of the clock gene Per2 exacerbates, whereas melatonin mitigates cholestatic liver injury in BDL mice.^7^^,^^30^, ^31^, ^32^ Our study revealed that BDL-induced changes in liver CE, BA, and serum C4 levels were accompanied by significant diurnal fluctuations, but not in FFA levels, indicating a link between the BA synthesis rhythm and cholestatic liver disease.

Additionally, BDL affected clock gene expression, with most genes showing reduced amplitude and loss of rhythmicity, except for Bmal1 and retinoic-acid-receptor-related orphan receptor-α (RORa), which presented slight phase shifts. As liver fibrosis progresses, the levels of Per1, Bmal1, Clock, and Cry1 increase in BDL rats,^9^ possibly due to strain and zeitgeber time differences. Further analysis revealed irregular circadian rhythms in CYP7A1 and key factors, such as Fxr and Shp, which are crucial for BA synthesis. The circadian clock's role in regulating BA synthesis is significant, with the clock genes Per1/Per2, Dbp, and Rev-erbα activating CYP7A1 in distinct but interrelated ways, potentially leading to cholestasis.^33^, ^34^, ^35^, ^36^ These findings suggest that circadian clock disturbances may contribute to cholestasis by affecting the rhythmic expression of BA synthesis genes.

The scavenger receptor CD36 facilitates fatty acid uptake and modulates lipid metabolism in both rodents and humans.^37^^,^^38^ Additionally, CD36 serves as a crucial regulator of cholesterol homeostasis and may activate LXR to increase BA synthesis from cholesterol and promote cholesterol excretion into bile.^39^ However, the association between CD36 and cholestatic liver disease has not yet been reported. One study indicated that hepatic NCOR1 deficiency modified the expression of BA synthesis genes and altered the circadian expression patterns of CD36.^40^ In our previous study, CD36 was identified as a vital regulator of the liver clock and the hepatic gluconeogenesis rhythm.^19^ To delve deeper into the role of CD36 in liver metabolic rhythms, we performed transcriptomic analysis and validation on the CD36 LKO mice. In alignment with previous results, diurnal variations in liver clock genes were absent in the CD36 LKO mice. Notably, the circadian expression of the BA synthesis genes CYP7A1 and HMGCR was significantly diminished, whereas there was no substantial effect on liver BA transporters. Furthermore, we observed that CD36 expression was elevated in the livers of patients with cholestasis. Consistently, CD36 levels were increased in the livers of BDL mice at various time intervals. These findings suggest that CD36 may be involved in the circadian regulation of hepatic BA synthesis, with its rhythmic expression potentially playing a significant role in cholestatic liver disease.

During cholestasis, serum and hepatic BA levels increase, leading to liver damage.^41^ Our data demonstrated that the liver-specific knockout of CD36 significantly reduced both liver and serum BA levels, as well as plasma C4 levels, while also improving liver inflammation and fibrosis in BDL mice. Intriguingly, circadian transcriptomics indicate that CD36 is involved in intricate and coordinated rhythmic regulation in cholestatic liver disease. Liver-specific deletion of CD36 reinstated the diurnal expression of 31 genes whose rhythmic expression was lost in BDL mice, including numerous genes involved in the core clock and energy metabolism. Additionally, cholestasis induced abnormal *de novo* rhythms in 1101 genes, whereas liver-specific deletion of CD36 maintained their expression in a normal pattern, including genes related to BA metabolism, such as HMGCR and CYP7A1. Given that cell interactions and crosstalk contribute to biliary fibrosis in cholestasis,^42^ we identified the primary cell classification of diurnally expressed genes. CD36 affects mainly the circadian expression of metabolism-related genes in hepatocytes but has a minimal impact on that of non-hepatic parenchymal cells, implying that the rhythmic regulation of hepatocytes by CD36 may also impact intercellular communication.

There is substantial evidence supporting a bidirectional relationship between BAs and circadian rhythms in metabolic regulation.^43^^,^^44^ Given the influence of liver CD36 on both systems, the potential mechanisms underlying the causal link between CD36, circadian rhythm, and BA homeostasis warrant further investigation. Studies have indicated that interventions such as melatonin administration and time-restricted feeding often lead to regular circadian gene expression and improved BA homeostasis, thereby alleviating cholestasis.^45^^,^^46^ Therefore, maintaining a normal circadian rhythm is crucial for driving BA oscillations. Circadian rhythms have evolved, in part, to synchronize feeding and fasting patterns. The loss of BA rhythmicity observed in BDL mice may result from a disruption in the food intake rhythm, as BAs are closely associated with feeding patterns.^43^ In our study, the activity and food intake of the mice following BDL surgery were significantly restricted, particularly during the night. Interestingly, hepatic-specific deletion of CD36 led to a marked recovery of these phenotypes, characterized by a resumption of normal diurnal behavioral rhythms with a preference for nocturnal feeding and activity (data not shown). Furthermore, the findings demonstrated that the deletion of CD36 in the liver mitigated BDL-induced circadian disruption of clock genes, suggesting that the impact of CD36 on BA circadian metabolism may be secondary to changes in clock gene oscillations.

In this study, the precise mechanisms by which CD36 influences the circadian clock remain inadequately explored. RNA sequencing and quantitative real-time PCR analyses revealed that BDL significantly reduced diurnal differences in Nr1d1, Nr1d2, and DBP expression while increasing those of HMGCR and CYP7A1. These alterations were partially reversed in the CD36 LKO mice. Although the correlation between DBP and HMGCR or CYP7A1 remains unclear, REV-ERBα is known to regulate key genes involved in cholesterol biosynthesis. REV-ERBα down-regulation up-regulates CYP7A1 and promotes BA metabolism, whereas REV-ERB agonists (*e.g.*, SR9011 and SR9009) reduce CYP7A1 expression,^47^^,^^48^ suggesting that REV-ERBα negatively regulates CYP7A1. Additionally, several studies have indicated that nuclear receptors are important molecular bases of metabolic feedback circadian clocks, some of which are regulated by CD36,^26^^,^^49^ suggesting the potential role of nuclear receptors in the regulation of clock genes by CD36. Given that REV-ERBα/β are members of the nuclear receptor superfamily, we hypothesize that CD36 may modulate diurnal BA synthesis via the REV-ERBα/CYP7A1 axis. However, further studies are needed to elucidate this mechanism.

The findings of this study indicate that CD36 exerts distinct regulatory effects on BA metabolism rhythms under physiological and pathological conditions. Low expression of CD36 can restore the liver clock of BDL mice to a certain extent, thereby improving disease progression. Lipid nanoparticles have revolutionized clinical nucleic acid delivery due to their enhanced biocompatibility, targeting efficiency, and reduced immunogenicity, paving the way for transformative therapies in gene silencing and mRNA vaccination.^50^^,^^51^ We used lipid nanoparticles with a controlled release advantage to inhibit CD36 and observed an improvement in the pathological phenotype of BDL mice. Cholestatic liver disease is characterized by dysregulated BA metabolism and disrupted circadian rhythms, highlighting the potential of therapies aimed at restoring these imbalances. Given the rhythmic oscillations of BA signaling, the efficacy of drugs can be enhanced by optimizing the timing of administration. Further research is needed to develop circadian rhythm-based therapeutic strategies for these conditions.

## CRediT authorship contribution statement

**Yang Zhang:** Project administration, Formal analysis, Writing – original draft, Investigation, Writing – review & editing, Methodology, Data curation. **Mingyang Zhang:** Writing – review & editing, Methodology, Data curation, Project administration, Formal analysis, Writing – original draft, Investigation. **Shuning Fu:** Methodology, Formal analysis, Investigation. **Zhenyu Wang:** Methodology, Formal analysis, Investigation. **Yunfei Zhao:** Software, Data curation, Methodology. **Junhua Gong:** Software, Data curation, Methodology. **Miao Chen:** Resources. **Nuo Zhang:** Visualization. **Mengyue Chen:** Writing – review & editing, Conceptualization, Funding acquisition. **Xiong Z. Ruan:** Funding acquisition, Supervision. **Yaxi Chen:** Funding acquisition, Supervision, Validation.

## Ethics declaration

Approval for all experiments involving human tissues was granted by the Ethics Committee of the Second Affiliated Hospital of Chongqing Medical University, and informed consent was obtained from all patients. The Ethics Committee of the Second Affiliated Hospital of Chongqing Medical University approved all the animal experiments (issue number: 2023215).

## Data availability

The data supporting the findings of this study are available from the corresponding author upon reasonable request.

## Funding

This work was supported by the National Natural Science Foundation of China (No. 32030054, 82170586, U23A20415, 32400943), the National Key R&D Program of China (No. 2022YFC2502500), the Science and Technology Research Program of Chongqing Municipal Education Commission (No. KJZD-K202200402, CSTB2023NSCQ-LZX0001), the Kuanren Talents Program of the Second Affiliated Hospital of Chongqing Medical University and Program for Youth Innovation in Future Medicine of Chongqing Medical University (No. W0163), the Postdoctoral Foundation of Chongqing Natural Science Foundation (No. TB2023NSCQ-BHX0145), and the Joint Project of Pinnacle Disciplinary Group of the Second Affiliated Hospital of Chongqing Medical University.

## Conflict of interests

Xiong Z. Ruan is an associate editor for *Genes & Diseases* and was not involved in the editorial review or the decision to publish this article. The remaining authors declare that they have no competing interests.
