# Supplementary material for: Elevated diurnal CD36 expression disrupts the bile acid synthesis rhythm leading to cholestatic liver injury and inflammation via the HMGCR/CYP7A1 axis
Source: Genes Dis. 2025 Jul 23;13(4):101776. doi: 10.1016/j.gendis.2025.101776 (PMC13091082; doi:10.1016/j.gendis.2025.101776)
Supplement: Multimedia component 1 [file mmc1.docx]

**Table S1. Detailed clinical features of PBC and PSC patients.**

| **Sex** | **Age (years)** | **Type** | **Pathology**  **diagnosis** | **ALP (IU/L)** | **GGT (IU/L)** | **TBA (μmol/L)** | **TBIL (μmol/L)** |
| --- | --- | --- | --- | --- | --- | --- | --- |
| M | 38 | PSC | Early-stage | 358 | 456 | 61.3 | 17.7 |
| M | 38 | PSC | Early-stage | 411 | 333 | 36.8 | 115.3 |
| M | 51 | PSC | Late-stage | 385 | 467 | 55.3 | 94.9 |
| M | 66 | PSC | Late-stage | 403 | 399 | 53.5 | 88.5 |
| F | 72 | PSC | Advanced-stage | 410 | 404 | 46.7 | 101 |
| F | 69 | PSC | Advanced-stage | 539 | 598 | 78.6 | 130.8 |
| M | 50 | PBC | Cholangitis | 560 | 546 | 86.5 | 168.3 |
| M | 46 | PBC | Cholangitis | 408 | 593 | 90.8 | 123.8 |
| M | 55 | PBC | Cholangitis | 453 | 307 | 70.1 | 112.8 |
| F | 61 | PBC | Cholangitis | 467 | 368 | 79.5 | 83.7 |
| M | 50 | PBC | Cholangitis | 445 | 351 | 77.2 | 91.9 |

**Note:** ALP, alkaline phosphatase; GGT, gamma-glutamyl transferase; TBA, total bile salts; TBIL, total bilirubin; PSC, primary sclerosing cholangitis; PBC, primary biliary cholangitis.

**Table S2. Information on Primer (human) Used in RT-qPCR. Related to STAR Methods.**

| **Gene** | **Upstream sequences（5'-3'）** | **Downstream sequences（5'-3'）** |
| --- | --- | --- |
| β-actin | GTTGTCGACGACGAGCG | GCACAGAGCCTCGCCTT |
| CD36 | TTCCTGCAGCCCAATGGT | TTGTCAGCCTCTGTTCCAACTG |

**Table S3. Information on Primer (mouse) Used in RT-qPCR. Related to STAR Methods.**

| **Gene** | **Upstream sequences（5'-3'）** | **Downstream sequences（5'-3'）** |
| --- | --- | --- |
| β-actin | CCTGAGGCTCTTTTCCAGCC | TAGAGGTCTTTACGGATGTCAACGT |
| Bmal1 | TGACCCTCATGGAAGGTTAGAA | GGACATTGCATTGCATGTTGG |
| Clock | ATGGTGTTTACCGTAAGCTGTAG | CTCGCGTTACCAGGAAGCAT |
| Per1 | GAATTGGAGCATATCACATCCGA | CCCGAAACACATCCCGTTTG |
| Per2 | CAGGTTGAGGGCATTACCTCC | AGGCGTCCTTCTTACAGTGAA |
| Per3 | AAAAGCACCACGGATACTGGC | GGGAGGCTGTAGCTTGTCA |
| Cry1 | CACTGGTTCCGAAAGGGACTC | CTGAAGCAAAAATCGCCACCT |
| Cry2 | CACTGGTTCCGCAAAGGACTA | CCACGGGTCGAGGATGTAGA |
| Rev-erbα | AACAGTCTACGGCAAGGCAA | GTGCTGAGAAAGGTCACGGA |
| Rorα | ATTAGGATGTGCCGTGCCTT | GCGATTTCGTCTTCGGTCAG |
| Dbp | GGATGGGTGGGTAGAATCATTGT | CGGGATCAGGTTCAAAGGTCATT |
| CD36 | ATGGGCTGTGATCGGAACTG | GTCTTCCCAATAAGCATGTCTCC |
| Cyp7a1 | AGCAACTAAACAACCTGCCAGTACTA | GTCCGGATATTCAAGGATGCA |
| Cyp7b1 | GCTCAGATGCAAGGACAGTCAAA | AGCCAAGATGATGTGCTCCTATT |
| Cyp8b1 | ACCTGTTTCTGGGTCCTCTTATT | ATAATCCTCCTGTACCACCCTGA |
| Cyp27a1 | GCTGAGGAAGAAAGAGGCTGATA | CCAGGGCAATCTCATACTTCTGT |
| Fxr | TCATCCTCTCTCCAGACAGACAA | ACTCTCCAAGACATCAGCATCTC |
| Lxr | AGAGAGGCTGCAACACACATATG | CTGAATGGACGCTGCTCAAAGTA |
| Nr0b2 | CACGATCCTCTTCAACCCAGATG | ATGTCAACGTCTCCCATGATAGG |
| Hmgcr | GTATTGCTGGCCTCTTCACAATTT | GATTGCCATTCCACGAGCTATAT |
| Bsep | GTTCTGAATGGACTGTCGGTATCT | TTTGCTGTCGTGACCATCTATCA |
| Mrp2 | TGTGGAAACTGGGAAGGTAAAGT | AGCCAGAGGTTAGTGCCAATAAA |
| Oatp1 | ACTATGCGTGGAATTGGTGAAAC | TCCAATAATCGGGCCAACAATCT |
| Ntcp | ATAGTGGCCCAGTACGGTATCAT | CATCACAATGCTGAGGTTCATGT |
| Lrh-1 | CATGGGAAGGAAGGGACAATCTT | GGTTCTCCAGGTTCTTCACATCT |
| Hnf4α | GCCAACCTCAATTCATCCAACAG | TCTACCACACATTGTCGGCTAAA |
| Abcg8 | ATCCGTCGTCAGATTTCCAATGA | CCGTCTTCCAGCTCATAGTACAG |
| Abcg5 | CCCAAGGTCATGATGCTAGATGA | CAGAACACCAACTCTCCGTAAGT |
| Il-1β | GCAACTGTTCCTGAACTCAACT | ATCTTTTGGGGTCCGTCAACT |
| Tnf-α | CCCTCACACTCAGATCATCTTCT | GCTACGACGTGGGCTACAG |
| Mcp1 | TTAAAAACCTGGATCGGAACCAA | GCATTAGCTTCAGATTTACGGGT |
| Il-6 | GCTACCAAACTGGATATAATCAGGAAA | CTTGTTATCTTTTAAGTTGTTCTTCATGTACTC |

**Table S4. Circadian rhythm parameters of genes in liver of SHAM and BDL mice.**

| **Gene** | **Group** | **P-value** | **Phase** | **Amplitude** | **Mesor** | **\|Δphase\|** | **ΔAMP** | **ΔMesor** |
| --- | --- | --- | --- | --- | --- | --- | --- | --- |
| Bmal1 | SHAM | 3.63E-09 | 22.3 | 0.54 | 0.55 | 1.4, P=0.081 | -0.06, P=0.553 | -0.08, P=0.23 |
|  | BDL | 1.40E-05 | 20.9 | 0.48 | 0.46 |  |  |  |
| Clock | SHAM | 4.77E-05 | 20.6 | 0.42 | 0.74 | 0.5, P=0.798 | -0.26, **P=0.007** | -0.41, **P<0.001** |
|  | BDL | 5.00E-03 | 20.1 | 0.15 | 0.33 |  |  |  |
| Per1 | SHAM | 2.92E-04 | 17 | 1.3 | 1.19 | / | / | / |
|  | BDL | 1.25E-01 | NR | NR | NR |  |  |  |
| Per2 | SHAM | 1.30E-03 | 15.1 | 5.23 | 4.73 | / | / | / |
|  | BDL | 8.50E-02 | NR | NR | NR |  |  |  |
| Per3 | SHAM | 9.33E-06 | 14.5 | 9.17 | 6.79 | 3.3, P=0.294 | -7.73, **P<0.001** | -4.99, **P<0.001** |
|  | BDL | 1.50E-03 | 11.2 | 1.44 | 1.8 |  |  |  |
| Cry1 | SHAM | 1.00E-02 | 18.2 | 0.42 | 0.52 | 0.6, P=0.863 | -0.3, P=0.045 | -0.32, P=0.004 |
|  | BDL | 4.00E-03 | 18.8 | 0.12 | 0.2 |  |  |  |
| Cry2 | SHAM | 4.00E-03 | 16.5 | 3.17 | 2.5 | 2.2, P=0.511 | -2.26, P=0.037 | -0.69, P=0.360 |
|  | BDL | 3.80E-02 | 18.7 | 0.91 | 1.82 |  |  |  |
| Rorα | SHAM | 1.15E-06 | 18.1 | 0.53 | 1.06 | 1.1, P=0.315 | 0.13, P=0.429 | -0.14, P=0.252 |
|  | BDL | 3.42E-04 | 19.2 | 0.66 | 0.92 |  |  |  |
| Dbp | SHAM | 3.48E-07 | 12.8 | 7.4 | 6.58 | 1.5, P=0.792 | -6.88, P=0.429 | -5.58, **P<0.001** |
|  | BDL | 9.40E-03 | 11.3 | 0.52 | 1 |  |  |  |
| Nr1d1 | SHAM | 1.96E-08 | 9.9 | 8.15 | 7.59 | / | / | / |
|  | BDL | 9.50E-02 | NR | NR | NR |  |  |  |
| Cyp7a1 | SHAM | 1.80E-02 | 17.9 | 0.48 | 1.05 | 3, P=0.186 | 1.08, **P=0.007** | 0.71, **P=0.011** |
|  | BDL | 1.39E-04 | 14.9 | 1.56 | 1.76 |  |  |  |
| Lxr | SHAM | 4.01E-07 | 18.9 | 0.69 | 1.51 | 0.9, P=0.536 | -0.36, **P=0.025** | -0.42, **P<0.001** |
|  | BDL | 1.20E-02 | 18 | 0.33 | 1.09 |  |  |  |
| Fxr | SHAM | 2.00E-03 | 16 | 0.4 | 1.22 | / | / | / |
|  | BDL | 7.92E-01 | NR | NR | NR |  |  |  |
| Shp | SHAM | 8.00E-03 | 15.5 | 1.01 | 2.31 | 0.9, P=0.536 | -0.49, P=0.211 | 1.25, **P<0.001** |
|  | BDL | 5.00E-03 | 21.9 | 0.52 | 1.06 |  |  |  |
| Bsep | SHAM | 6.02E-04 | 2.8 | 0.23 | 0.95 | / | / | / |
|  | BDL | 4.14E-01 | NR | NR | NR |  |  |  |
| Mrp2 | SHAM | 3.00E-03 | 18.6 | 0.32 | 0.82 | / | / | / |
|  | BDL | 1.29E-01 | NR | NR | NR |  |  |  |

**Table S5. Circadian rhythm parameters of genes in liver of CD36^fl/fl^ and CD36 LKO mice.**

| **Gene** | **Group** | **P-value** | **Phase** | **Amplitude** | **Mesor** | **\|Δphase\|** | **ΔAMP** | **ΔMesor** |
| --- | --- | --- | --- | --- | --- | --- | --- | --- |
| Bmal1 | CD36^fl/fl^ | 1.17E-08 | 23.7 | 0.5 | 0.58 | 2.2, **P=0.046** | 1.21, **P<0.001** | 0.70, **P<0.001** |
|  | CD36LKO | 4.26E-09 | 1.9 | 1.71 | 1.28 |  |  |  |
| Clock | CD36^fl/fl^ | 3.70E-02 | 0.2 | 0.15 | 0.88 | 0.7, P=0.841 | 1.18, **P<0.001** | 1.32, **P<0.001** |
|  | CD36LKO | 2.65E-07 | 0.9 | 1.33 | 2.2 |  |  |  |
| Per1 | CD36^fl/fl^ | 1.29E-04 | 11.4 | 2.02 | 3.85 | 3.5, **P=0.045** | -1.15, **P=0.030** | -1.26, **P=0.001** |
|  | CD36LKO | 4.10E-03 | 7.9 | 0.87 | 2.59 |  |  |  |
| Per2 | CD36^fl/fl^ | 5.14E-05 | 14.1 | 1.33 | 1.99 | 3.2, **P=0.008** | 0.14, P=0.737 | -0.05, P=0.876 |
|  | CD36LKO | 1.88E-04 | 10.9 | 1.47 | 1.95 |  |  |  |
| Per3 | CD36^fl/fl^ | 3.46E-09 | 12.2 | 4.85 | 4.63 | 2.4, **P<0.001** | -0.18, P=0.812 | 1.13, **P=0.039** |
|  | CD36LKO | 4.43E-08 | 9.8 | 4.67 | 5.76 |  |  |  |
| Cry1 | CD36^fl/fl^ | 2.54E-06 | 18.7 | 0.52 | 0.99 | 4.7, **P<0.001** | 0.06, P=0.719 | 0.10, P=0.428 |
|  | CD36LKO | 4.21E-04 | 23.4 | 0.58 | 1.09 |  |  |  |
| Cry2 | CD36^fl/fl^ | 3.06E-04 | 11.4 | 1.74 | 2.92 | 9.6, **P<0.001** | -0.10, P=0.898 | 2.63, **P<0.001** |
|  | CD36LKO | 3.45E-02 | 1.8 | 1.64 | 5.55 |  |  |  |
| Rorα | CD36^fl/fl^ | 3.45E-02 | 6.4 | 0.44 | 1.52 | 7.0, **P=0.049** | 0.93, P=0.092 | 3.26, **P<0.001** |
|  | CD36LKO | 1.25E-02 | 23.4 | 1.37 | 4.78 |  |  |  |
| Dbp | CD36^fl/fl^ | 3.46E-05 | 11.4 | 1.74 | 2.92 | / | / | / |
|  | CD36LKO | 6.51E-01 | NR | NR | NR |  |  |  |
| Nr1d1 | CD36^fl/fl^ | 1.34E-05 | 8.3 | 50.13 | 32.64 | 0.9, P=0.473 | -26.14, **P=0.009** | 7.39, P=0.277 |
|  | CD36LKO | 4.46E-07 | 9.2 | 23.99 | 25.25 |  |  |  |
| Cyp7a1 | CD36^fl/fl^ | 1.02E-05 | 19.3 | 0.5 | 1.3 | 10.3, **P<0.001** | -0.21, P=0.142 | -0.64, **P<0.001** |
|  | CD36LKO | 2.63E-02 | 9 | 0.29 | 0.66 |  |  |  |
| Cyp8b1 | CD36^fl/fl^ | 1.38E-02 | 23.4 | 0.15 | 0.8 | 0.4, P=0.942 | 1.65, **P<0.001** | 1.03, **P<0.001** |
|  | CD36LKO | 2.28E-05 | 23 | 1.8 | 1.83 |  |  |  |
| Cyp7b1 | CD36^fl/fl^ | 9.43E-04 | 11.6 | 1.15 | 2.41 | 2.6, P=0.097 | -0.15, P=0.730 | 0.87, **P=0.007** |
|  | CD36LKO | 5.56E-03 | 9 | 1 | 3.28 |  |  |  |
| Cyp27a1 | CD36^fl/fl^ | 3.96E-02 | 10.4 | 0.35 | 1.35 | / | / | / |
|  | CD36LKO | 5.56E-03 | NR | NR | NR |  |  |  |
| Fxr | CD36^fl/fl^ | 4.24E-02 | 18.9 | 0.2 | 0.81 | 0.1, P=0.966 | 0.17, P=0.224 | 0.01, P=0.845 |
|  | CD36LKO | 1.03E-03 | 19 | 0.37 | 0.82 |  |  |  |
| Lxr | CD36^fl/fl^ | 6.58E-03 | 10.3 | 0.38 | 1.08 | / | / | / |
|  | CD36LKO | 1.03E-03 | NR | NR | NR |  |  |  |
| Nr0b2 | CD36^fl/fl^ | 3.41E-02 | 9.5 | 1.53 | 3.71 | 2.9, P=0.211 | -0.38, P=0.629 | -1.03, P=0.067 |
|  | CD36LKO | 5.12E-03 | 6.6 | 1.15 | 2.68 |  |  |  |
| Hmgcr | CD36^fl/fl^ | 6.70E-04 | 20.7 | 0.44 | 0.63 | 12.4, **P<0.001** | -0.28, **P=0.017** | -0.41, P=5.719 |
|  | CD36LKO | 1.52E-04 | 8.3 | 0.16 | 0.22 |  |  |  |
| Bsep | CD36^fl/fl^ | 1.73E-03 | 4.8 | 0.3 | 1.13 | 0.1, P=0.944 | 0.04, P=0.784 | 0.17, P=0.084 |
|  | CD36LKO | 1.52E-04 | 4.9 | 0.34 | 1.3 |  |  |  |
| Mrp2 | CD36^fl/fl^ | 4.86E-03 | 12.4 | 0.39 | 1.41 | 1.5, P=0.334 | 0.05, P=0.756 | 0.19, P=0.111 |
|  | CD36LKO | 6.98E-04 | 10.9 | 0.44 | 1.6 |  |  |  |
| Oatp1 | CD36^fl/fl^ | 4.26E-02 | 9.8 | 0.13 | 1 | 2.6, P=0.274 | 0.05, P=0.608 | -0.02, P=0.742 |
|  | CD36LKO | 1.86E-02 | 7.2 | 0.18 | 0.98 |  |  |  |
| Ntcp | CD36^fl/fl^ | 7.54E-03 | 11.6 | 0.28 | 1.22 | 1.2, P=0.414 | 0.05, P=0.661 | -0.11, P=0.178 |
|  | CD36LKO | 2.26E-05 | 12.8 | 0.33 | 1.11 |  |  |  |
| Lrh-1 | CD36^fl/fl^ | 2.70E-07 | 10.1 | 1.86 | 3.5 | 1.6, P=0.137 | -0.61, P=0.122 | 0.02, P=0.935 |
|  | CD36LKO | 3.52E-04 | 8.5 | 1.25 | 3.52 |  |  |  |
| Hnf4α | CD36^fl/fl^ | 5.04E-04 | 19 | 0.81 | 1.85 | 2.2, P=0.146 | -0.24, P=0.356 | 0.08, P=0.671 |
|  | CD36LKO | 2.18E-03 | 21.2 | 0.57 | 1.93 |  |  |  |
| Abcg8 | CD36^fl/fl^ | 8.81E-07 | 9.6 | 2.73 | 3.58 | 0, P=0.965 | -0.53, P=0.288 | 0.55, P=0.121 |
|  | CD36LKO | 1.37E-07 | 9.6 | 2.2 | 4.13 |  |  |  |
| Abcg5 | CD36^fl/fl^ | 9.19E-04 | 7.2 | 1.44 | 2.28 | 1.1, P=0.489 | -0.33, P=0.518 | 0.85, **P=0.022** |
|  | CD36LKO | 4.05E-03 | 6.1 | 1.11 | 3.13 |  |  |  |
